# GSA STUDENT CHAPTER AS A GATEWAY TO UNDERGRADUATE STUDENT UNDERSTANDING OF PROFESSIONAL ORGANIZATION INVOLVEMENT

**DOI:** 10.1093/geroni/igae098.1325

**Published:** 2024-12-31

**Authors:** Lauren Bouchard

**Affiliations:** Western Oregon University, Salem, Oregon, United States

## Abstract

The Portland State University Student Chapter has created networking events for students across Oregon, and undergraduate students are especially important to the chapter. Due to the GSA annual meeting in Seattle, our chapter had the unique opportunity to educate students across the Pacific Northwest as to the importance of professional organization membership. In partnership with the Oregon Gerontological Association (OGA), Western Oregon University (WOU), and Portland Community College (PCC), our chapter created an educational event regarding the structure, events, and benefits of GSA. Undergraduate students may differ in their knowledge and understanding of opportunities they perceive to be beyond their educational level. For this reason, our Careers in Aging Month (CIAM) event focused on membership in the larger GSA organization with support from the Emerging Scholars Professional Organization (ESPO) section within GSA. We highlighted potential differences for undergraduate students, including the new change that allows these students to gain GSA membership for free. This presentation will explain chapter activities designed to encourage undergraduate student membership as well as provide suggestions to recruit and engage undergraduate student members.

